# Humic substance-mediated reduction of iron(III) oxides and degradation of 2,4-D by an alkaliphilic bacterium, *Corynebacterium humireducens* MFC-5

**DOI:** 10.1111/1751-7915.12003

**Published:** 2012-12-06

**Authors:** Chun-yuan Wu, Li Zhuang, Shun-gui Zhou, Yong Yuan, Tian Yuan, Fang-bai Li

**Affiliations:** 1 Guangdong Key Laboratory of Agricultural Environment Pollution Integrated Control, Guangdong Institute of Eco-Environmental and Soil SciencesGuangzhou, 510650, China; 2 Institute of Environment and Plant Protection, Chinese Academy of Tropical Agricultural SciencesHaikou, 571101, China

## Abstract

With the use of an alkaliphilic bacterium, *Corynebacterium humireducens* MFC-5, this study investigated the reduction of goethite (α-FeOOH) and degradation of 2,4-dichlorophenoxyacetic acid (2,4-D) mediated by different humic substances (humics) and quinones in alkaline conditions (pH of 9.0). The results indicated that (i) using sucrose as the electron donor, the strain MFC-5 was capable of reducing anthraquinone-2,6-disulfonic acid (AQDS), anthraquinone-2-disulfonic acid (AQS), anthraquinone-2-carboxylic acid (AQC), humic acid (HA) and fulvic acid (FA), and its reducing capability ranked as AQC > AQS > AQDS > FA > HA; (ii) the anaerobic reduction of α-FeOOH and 2,4-D by the strain was insignificant, while the reductions were greatly enhanced by the addition of quinones/humics serving as redox mediators; (iii) the Fe(III) reduction rate was positively related to the content of quinone functional groups and the electron-accepting capacities (EAC) of quinones/humics based on fourier-transform infrared spectroscopy (FT-IR) and electrochemical analyses; however, such a relationship was not found in 2,4-D degradation probably because quinone reduction was not the rate-limiting step of quinone-mediated reduction of 2,4-D. Using the example of α-FeOOH and 2,4-D, this study well demonstrated the important role of humics reduction on the Fe(III)/Fe(II) biogeochemical cycle and chlorinated organic compounds degradation in alkaline reducing environments.

**Funding Information** This study was supported by the National Natural Science Foundation of China (Nos 41101211, 31070460, 41101477), and The Project Sponsored by the Scientific Research Foundation for the Returned Overseas Chinese Scholars, State Education Ministry.

## Introduction

Humic substances (humics) are polymeric, heterogeneous redox-active natural organic compounds and are ubiquitous in soils, sediments, and natural waters (Stevenson, 1994). The finding of humics as terminal electron acceptors for microbial respiration (Lovley *et al*., 1996) reveals the important role of humics in many biogeochemical cycles happened in soils and aquatic sediments. Reduced humics can transfer electrons abiotically to terminal electron acceptors such as high valence metals (Gu and Chen, 2003) or oxidized organic contaminants (Curtis and Reinhard, 1994; Fu *et al*., 1999), functioning as electron shuttles between microorganisms and terminal electron acceptors. The redox-mediating ability of humics thus affects the cycling of redox-active elements (e.g. Fe, Mn) and the transformation of organic/inorganic contaminants in suboxic or anoxic systems.

Humics in environments can be categorized into three main fractions as humic acids (HA), fulvic acids (FA) and humin. The soluble constituent of humics is strongly pH-dependent (Stevenson, 1994): humin is the fraction insoluble at all pH values, HA constitutes the fraction soluble at pH > 2, while FA is soluble at all pH values. Thus, a high pH would increase the fraction of dissolved humics that are accessible for bacterial humics respiration. Humics-reducing microorganisms are phylogenetically diverse, including Fe(III)-reducing (Lovley *et al*., 1996), fermenting (Benz *et al*., 1998), sulfate-reducing and methanogenic bacteria (Cervantes *et al*., 2002). They are isolated and described from a broad diversity of environments, mainly with circumneutral pH (Lovley *et al*., 1996). Recently, researchers have turned their attention to finding humics-reducing microorganisms in alkaline environments, and several microbes (e.g. *Alkaliphilus peptidofermentans*, *Bacillus pseudofirmus*, *Natronincola ferrireducens*, *Natronincola peptidovorans*) have been isolated from alkaline environments (Zhilina *et al*., 2009a,2009b; Ma *et al*., 2012). In our recent work, a halotolerant, alkaliphilic, humics-reducing bacterium, belonging to the genus *Corynebacterium* was isolated from a microbial fuel cell (MFC) that was continuously fed with artificial wastewater of pH 10.0 (Wu *et al*., 2011). The identification of this alkaliphilic microorganism expanded the pH limit to 11.0 for microbial humics reduction, and also implicated the potential contribution of microbe-mediated humics reduction in natural alkaline environments.

Soda lakes and soda deserts are the most representative naturally occurring alkaline environments. Artificially generated alkaline environments could be derived from diverse industrial activities such as cement manufacture, alkaline electroplating, leather tanning and herbicide manufacture (Ulukanli and Diğrak, 2002 and references therein). For example, a field site from a former herbicide production plant that was heavily contaminated with organochlorines generated an aqueous pH up to 12 (Müller *et al*., 1999). The mineralogy and geochemistry of alkaline systems are highly complex and cannot be simply deduced from those studies conducted under neutral or acidic conditions. For instance, as iron(III) oxides are generally insoluble, solid-phase minerals under neutral or alkaline conditions, iron(III) reduction had not been recognized to occur above a pH of 9.0 before a alkaliphilic bacterium capable of iron(III) reduction was isolated from alkaline environment (Ye *et al*., 2004). Given the favourable solubility of humics under alkaline conditions, the contribution of humics will become ever more important to redox reactions under alkaline conditions.

To investigate the probable impact of microbial humics reduction on redox reactions in alkaline environments, *C. humireducens* MFC-5, a bacterium capable of humics reduction at pH as high as 11.0 and salinity of 12%, was used in alkaline anaerobic incubation. Humics-mediated reductive transformation of high valence metals and organic contaminants was conducted with the example of goethite (α-FeOOH) and 2,4-dichlorophenoxyacetic acid (2,4-D) respectively. This study aimed to investigate: (i) the ability of the strain MFC-5 to reduce humics, iron(III) oxides and 2,4-D under alkaline conditions, (ii) the reductive transformation of iron(III) oxides and 2,4-D coupled to the humics reduction by *C. humireducens* MFC-5, and (iii) the mechanism of electron transfer behind the humics-mediated catalytic reduction of iron(III) oxides and 2,4-D by *C. humireducens* MFC-5.

## Results and discussion

### Alternative electron acceptors

**Figure 1:**
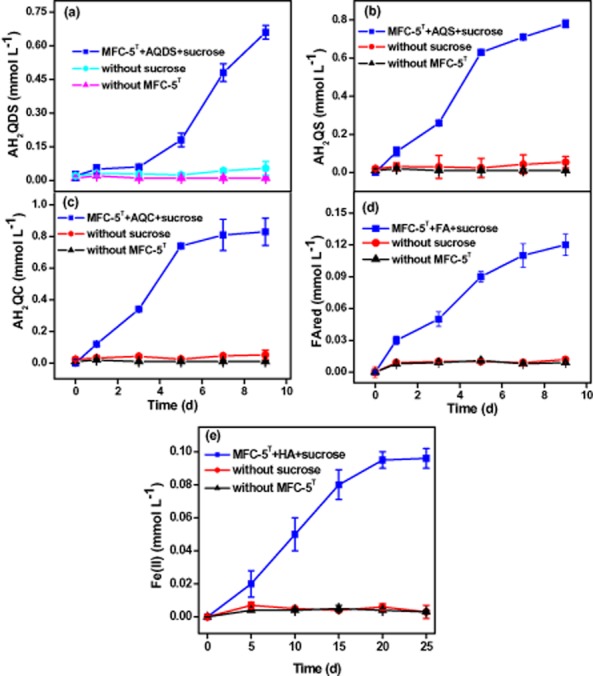
The reduction of quinones and humics by *C. humireducens* MFC-5 with sucrose as the electron donor: (a) AQDS; (b) AQS; (c) AQC; (d) FA; (e) HA. The experiments were performed under anaerobic conditions at 30°C for 10 days. Error bars represent standard deviation of the mean (*n* = 3).

Here we investigated the ability of *C. humireducens* MFC-5 to conserve energy to support cell growth with quinones [anthraquinone-2,6-disulfonic acid (AQDS), anthraquinone-2-sulfonic acid (AQS) and anthraquinone-2-carboxylic acid (AQC)] or humics (FA or HA) serving as the electron acceptor under alkaline conditions. Figure 1 presents the kinetics of microbial quinones/humics reduction by strain MFC-5 using sucrose as the electron donor at pH 9.0. After a 10-day incubation, the concentration of quinones/humics in the controls lacking sucrose (biotic control) or active cells (abiotic control) remained almost unchanged, demonstrating that quinones/humics were persistent in the absence of microbial activity of MFC-5 and the chemical reduction of quinones/humics by sucrose was negligible. In contrast, quinones/humics were significantly reduced in the active treatments (sucrose + quinones/humics + MFC-5), and the reduced AQDS, AQS, AQC and FA (AH_2_QDS, AH_2_QS, AH_2_QC and FAred) reached 0.66 ± 0.03, 0.78 ± 0.02, 0.82 ± 0.09 and 0.12 ± 0.01 mmol l^−1^ respectively. The amount of reduced HA (HAred) was determined by adding Fe(III) and determining how much Fe(II) was produced (Lovley *et al*., 1996), and approximately 0.096 ± 0.006 mmol l^−1^ of Fe(II) was produced in the treatments containing sucrose and MFC-5 after a 25-day incubation. In other words, the microequivalents involved in the microbial reduction of AQDS, AQS, AQC, FA and HA by strain MFC-5 were to be 1.32, 1.56, 1.64 and 0.12 and 0.096 eq l^−1^ respectively. These results suggested that (i) AQDS, AQS, AQC, FA and HA were able to serve as favourable electron acceptors in the anaerobic metabolism of strain MFC-5 under alkaline conditions, and (ii) the reducing capability of strain MFC-5 for quinones/humics was ranked as AQC > AQS > AQDS > FA > HA.

### Humics-enhanced Fe(III) reduction by strain MFC-5

**Figure 2:**
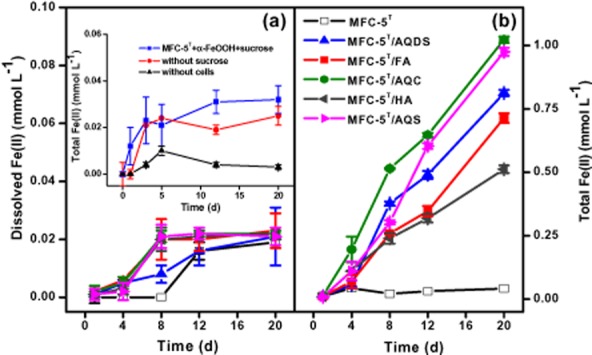
Production of Fe(II) during the bioreduction of 5 mmol l^−1^ α-FeOOH by *C. humireducens* MFC-5 provided with 5 mmol l^−1^ sucrose as the electron donor and in the presence of electron transfer mediators (quinones or humics): (a) dissolved Fe(II); (b) total Fe(II). Inset shows total Fe(II) production from α-FeOOH reduction by MFC-5 with sucrose serving as the electron donor (without sucrose or active cells as control). The experiments were performed under anaerobic conditions at 30°C for 20 days. Error bars represent standard deviation of the mean (*n* = 3).

Using a similar experimental procedure, microbial reduction of iron(III) oxides by *C. humireducens* MFC-5 was tested with a synthetic poorly crystalline iron(III) oxide, α-FeOOH. However, almost no Fe(III) reduction was observed in all the treatments (inset figure of Fig. 2a), suggesting that the strain MFC-5 can not perform Fe(III) reduction coupled to sucrose oxidation. Though all Fe(III)-reducing microorganisms are capable of using humics as electron acceptor (Lovley *et al*., 1996; 1998; Coates *et al*., 1998), the ability to reduce extracellular quinones of humics-reducing microorganisms is not always directly related to their ability to reduce iron(III) oxides. This is the case for the strain *C. humireducens* MFC-5 in this study. However, the addition of quinones/humics (AQDS, AQS, AQC, FA or HA) enhanced the rate and extent of α-FeOOH reduction significantly. Data showed that the total Fe(II) concentration after 20 days of incubation increased from 0.03 mmol l^−1^ in the active culture without quinones/humics to 0.81, 0.98, 1.02, 0.71 and 0.51 mmol l^−1^ in the active cultures with the amendment of AQDS, AQS, AQC, FA and HA respectively (Fig. 2b). For all treatments, the dissolved Fe(II) accounted for only a very small fraction of the total Fe(II) (Fig. 2a), which was expected because alkaline pH could result in precipitation of iron as Fe(OH)_3_.

The stimulation effects of quinones/humics on Fe(III) reduction are believed to be related to the microbial processes of quinones/humics since α-FeOOH was persistent in the abiotic control containing α-FeOOH and quinones/humics but without microbial activity of strain MFC-5. The ability of quinones/humics to stimulate Fe(III) reduction followed a order of AQC > AQS > AQDS > FA > HA, which was exactly consistent with the above reported capability of MFC-5 to reduce quinones/humics. Kappler and colleagues (2004) have suggested that the electron transfer through microbial reduction of humics represents an important path of electron flow in anoxic environments. In the mixed culture, the exogenous soluble redox active compounds (AQDS, AQS, AQC, FA and HA) were biologically reduced by MFC-5 and their reduced form (e.g. AH_2_QDS for AQDS) abiotically transferred electrons to α-FeOOH where they were reoxidized and again served as electron acceptors for MFC-5. The cycle would continue until sucrose, α-FeOOH or other necessary nutrients were depleted. This route has been proposed as an important mechanism for the enhancement of iron(III) oxides reduction by alleviating the need for direct contact between the cell and the oxides surface (Lovley *et al*., 1996; 1998; Scott *et al*., 1998; Zachara *et al*., 1998).

**Figure 3:**
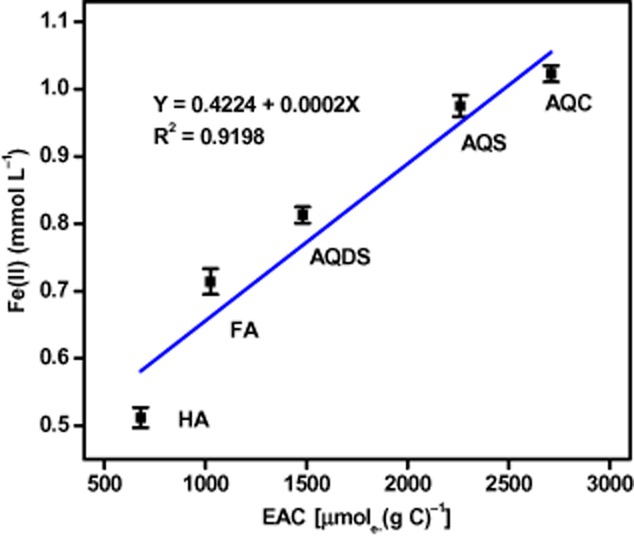
Linear correlation of total Fe(II) produced from α-FeOOH reduction with EAC of five types of electron shuttles in the α-FeOOH/MFC-5/humics (quinones)/sucrose system. Error bars represent standard deviation of the mean (*n* = 3).

The influence of humics on iron(III) oxides reduction has been extensively studied using AQDS as a surrogate for humics (Zachara *et al*., 1998; Nevin and Lovley, 2002; Liu *et al*., 2007; Wolf *et al*., 2009). This study investigated the effects of aquatic FA, HA, and quinones with different chemical structures and redox potentials on Fe(III) reduction, and they exhibited different influence on α-FeOOH reduction by MFC-5. Majority studies agreed that quinone functional groups are the main important electron-accepting and shuttling moieties in humics (Lovley *et al*., 1996; Scott *et al*., 1998). To find out the reason for the different stimulation effects, FT-IR was used to investigate the difference of quinone groups in AQDS, AQS, AQC, FA and HA. In the FT-IR spectrum, the absorption at about 1650 and 1630 cm^−1^ can be assigned to the C=O stretching of quinones (D'Orazio and Senesi, 2009). If the intensity of a band can be measured as the percent transmittance of the IR radiation, the amount of quinones groups in the redox-active compounds can be ranked as AQC > AQS > AQDS > FA > HA (Fig. S1). Employing an electrochemical approach (chronoamperometry) established by Yuan and colleagues (2011), the electron-accepting capacities (EAC) of AQDS, AQS, AQC, FA and HA were determined to be 1481, 2261, 2710, 1025 and 680 μmol_e−_ (g c)^−1^ respectively. Thus, the order of ability of accepting electrons was AQC > AQS > AQDS > FA > HA, which well agreed with the sequence of quinone group content in these quinones/humics as determined by FT-IR. Plotting the EAC of the different quinones/humics versus Fe(II) produced in the quinones/humics-supplemented cultures, a high positive correlation (R^2^ = 0.92) was established (Fig. 3). A conclusion can be drawn that the extent of enhancement in Fe(III) reduction induced by quinones/humics was highly dependent on the amount of quinone groups that were responsible for the electron accepting and shuttling properties in the redox active compounds. Our results are similar with Wolf's study which evidenced that the kinetics of microbial iron reduction mediated by quinones was primarily depended on the redox potential of the shuttle compound (Wolf *et al*., 2009).

### Humics-enhanced 2,4-D anaerobic degradation by strain MFC-5

**Figure 4:**
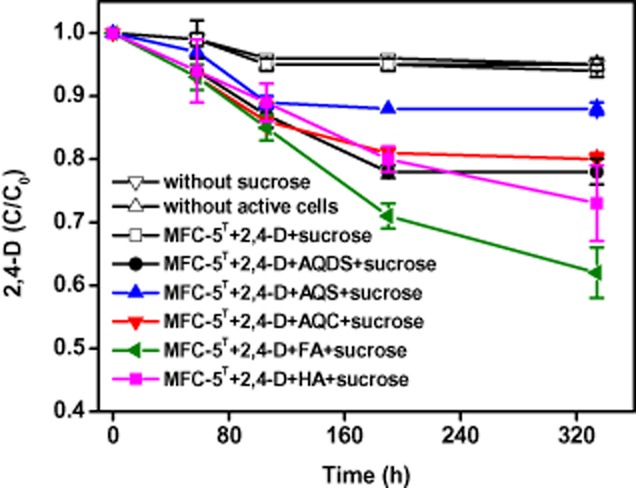
The transformation rate of 2,4-D in the MFC-5/humics (quinones) system with sucrose as the electron donor. Three types of treatments served as controls: MFC-5 + 2,4-D + sucrose, MFC-5 + 2,4-D, 2,4-D + sucrose + dead cells. The experiments were performed under anaerobic conditions at 30°C for 16 days. Error bars represent standard deviation of the mean (*n* = 3).

Further, the reductive degradation of 2,4-D by strain MFC-5 under alkaline conditions was studied. As shown in Fig. 4, for the treatments of 2,4-D + MFC-5, 2,4-D + sucrose, and 2,4-D + sucrose + MFC-5, approximately 5% of 2,4-D was reduced within 106 h and no further decrease in 2,4-D concentration was observed after that. The results were pretty similar to Fe(III) reduction by MFC-5 and suggested that there was only slight stimulation of MFC-5 on 2,4-D reduction. The amendment of AQDS, AQS, AQC, FA and HA in the system of 2,4-D + sucrose + MFC-5 led to a significant decrease in 2,4-D, and achieved a final removal rate of 22.1%, 12.2%, 19.4%, 38.7% and 26.4% within 330 h respectively. According to the literature (Walters, 1999), there are two anaerobic aquatic metabolisms for 2,4-D degradation: (i) 2,4-D → 2,4-dichlorophenol (2,4-DCP) → 4-chlorophenol (4-CP), and (ii) 2,4-D → 2,4-DCP → 2,4-dichloroanisole (2,4-DCA). In the anaerobic incubation of 2,4-D + sucrose + MFC-5 + quinones/humics, 2,4-DCP was observed as the intermediate metabolic product, and high-performance liquid chromatography (HPLC) analysis ruled out the existence of 4-CP but not 2,4-DCA. These results likely suggested that 2,4-D degraded through pathway (ii) rather than metabolism (i), which was generally proposed for anaerobic degradation under neutral pH conditions (Boyle *et al*., 1999; Berestovskaya *et al*., 2000).

**Figure 5:**
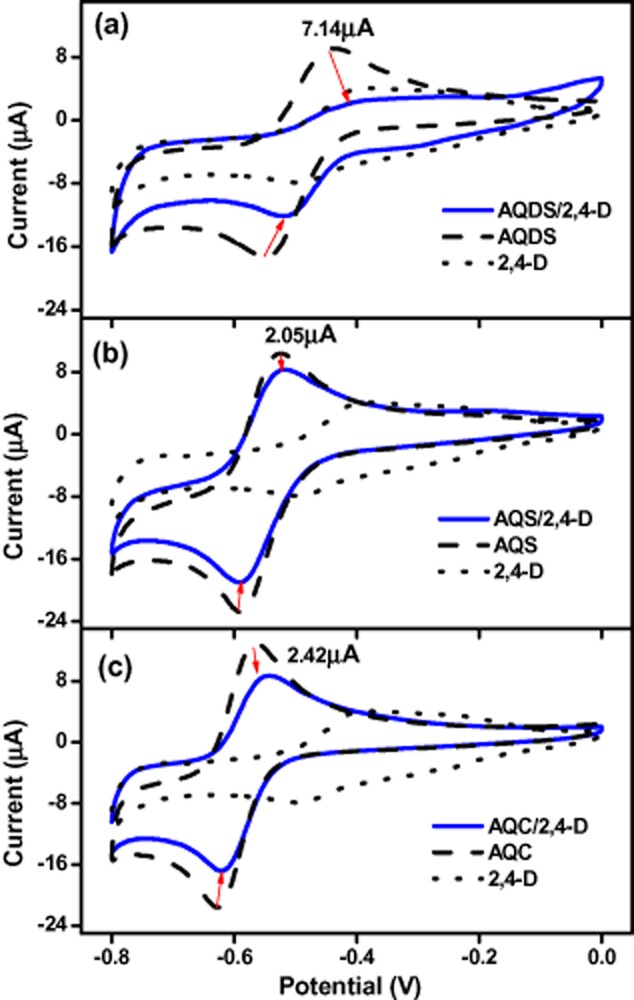
Cyclic voltammograms of quinones, 2,4-D and 2,4-D/quinones under aqueous conditions. (a) AQDS; (b) AQS; (c) AQC. Working electrode, Pt disk (3 mm diameter); counter electrode, Hg wire; reference electrode, Ag/AgCl; 500 mmol l^−1^ NaClO_4_ electrolyte; scan rate, 50 mVs^−1^.

2,4-D was degraded in the active culture containing 2,4-D, sucrose, MFC-5 and quinones/humics. In this system, microbial respiration of quinones/humics by strain MFC-5 should have occurred. The possibility of 2,4-D biodegradation by MFC-5, abiotic reduction of 2,4-D by sucrose or dead cell had been eliminated, it could be deduced that 2,4-D reduction was a consequence of quinones/humics respiration by MFC-5. As quinones/humics were reduced by MFC-5, reduced quinones/humics were generated and could provide electrons that were required for 2,4-D reductive degradation. To explore the electron transfer between reduced quinones/humics and 2,4-D, cyclic voltammetry (CV) was used to evaluate redox reaction in the system of quinones/humics and 2,4-D. Because of the different solvents used in the three-electrode cell for 2,4-D/quinones (aqueous) and 2,4-D/humics (organic), the CVs of quinones and humics cannot provide a meaningful comparison. Figure 5 displays the CVs recorded for quinones, 2,4-D and quinones + 2,4-D. CVs of AQDS, AQS, AQC exhibited well defined cathodic and anodic peaks corresponding to the reduction of quinone moieties and the oxidation of their reductive products produced at the electrode surface. Voltammograms of the mixture all showed that the redox couple potentials shifted to a more positive value as compared with the CV of the individual quinones, suggesting electron transfer between quinones and 2,4-D. It is obvious that AQDS suffered the greatest change of the peak current, followed by AQC and AQS, implying that more electrons are transferred from AQDS to 2,4-D. This electrochemical analysis supported the order of 2,4-D removal: AQDS > AQC > AQS. Different from stimulated Fe(III) reduction by quinones, the rate and extent of 2,4-D degradation enhanced by quinones was not directly related to the quinone group content and the EAC of quinones (AQC > AQS > AQDS). Though the electrochemical quantification (EAC, CVs in Fig 5) in this study all suggested that compared with AQDS, AQC and AQS are more effective redox mediators, the fact was that AQDS more effectively increased the degradation rate of 2,4-D than other quinone compounds. These results implied that quinone reduction might not be the rate-limiting step of quinone-mediated reduction of 2,4-D. Two independent reaction steps have been previously proposed for quinone-mediated reduction of azo dyes, consisting of quinones reduction by quinone reductase generating hydroquinones and further reductive azo bond cleavage by hydroquinone (Rau and Stolz, 2003). A similar two independent reaction steps were believed to be involved in quinone-mediated degradation of 2,4-D, and the second step of chemical redox reaction might be crucial to regulate the rate and extent of 2,4-D reductive degradation. Further research is underway in our laboratory to investigate the rate-limiting step of quinone-mediated reduction of iron(III) oxides and 2,4-D.

### Environmental implications

The genus *Corynebacterium* represents a large group of Gram-positive, asporogenous, rod-shaped bacteria with a high DNA G + C content (Liebl, 2010), and now includes over 100 species. Among them, there are less than 10 strains capable of growing under alkaline conditions, including *C. halotolerans* (Chen *et al*., 2004), *C. marinum* (Du *et al*., 2010), *C. maris* (Ben-Dov *et al*., 2009), *C. matruchotii* (Barrett *et al*., 2001), *C. terpenotabidum* (Collins *et al*., 2001), etc. *Corynebacterium humireducens* MFC-5 was thus far the only alkaliphilic *Corynebacterium* species capable of reducing quinones/humics. Driven by MFC-5, quinones/humics could function as electron shuttles to transfer electrons in redox reactions between sucrose and α-FeOOH or 2,4-D in alkaline environments (Fig. S2).

The redox cycling of iron plays a critical role in a range of biogeochemical processes in anoxic soils and aquifers, reactive Fe(II) species are important reductants for the abiotic transformation of many organic pollutants such as chlorinated contaminants, nitroaromatic explosive, and pesticides (Williams *et al*., 2005; Kim and Strathmann, 2007; Wu *et al*., 2010). At a circumneutral pH, Fe(II) species are generally produced via microbial iron reduction coupled to the oxidation of organic matter by dissimilatory Fe(III)-reducing microorganisms (Lovley *et al*., 2004; Weber *et al*., 2006). Due to the decrease of aqueous Fe(III) and increase of aqueous humics under alkaline conditions, the bioavailability of terminal electron acceptor for Fe(III)-reducing microorganisms becomes lower than that for humics-reducing bacteria. Driven by the activity of alkaliphilic bacteria, such as MFC-5 in this study, humics reduction would promote Fe(II) accumulation in alkaline environments, consequently affecting Fe(II)-associated redox reactions. Considering the large mass of humics in natural environments, humics-mediated Fe(III) reduction might be the predominant source of Fe(II) species over microbial metabolism.

Functioning as electron shuttles, humics have been shown to abiotically catalyse the reductive degradation of various xenobiotics (Van der Zee and Cervantes, 2009), for example, 2,4-D in our study. Theoretically, electron transfer from organic matter towards pollutants can occur provided that the standard redox potential of the electron shuttle is in between those of the two eventual half reactions as described in Fig. S2. Thus, humics reduction by alkaliphilic humics-reducing microorganisms might provide an effective remediation strategy for recalcitrant contaminants under alkaline environments.

### Conclusion

The alkaliphilic and halotolerant bacterium, *C. humireducens* MFC-5, could conduct anaerobic metabolism with quinones/humics as the sole terminal electron acceptor coupled to sucrose oxidation, but failed to perform anaerobic reduction of iron(III) oxides or 2,4-D. The presence of quinones/humics can significantly enhance α-FeOOH reduction and 2,4-D anaerobic degradation by MFC-5 under alkaline conditions. Quinone molecule acted as redox mediator which was biotically reduced by strain MFC-5, and reduced quinones/humics abiotically transferred electrons to exogenous Fe(III) or 2,4-D. The rate of Fe(II) production was shown to be positively related to the quinone group content and the EAC of the mediators, but not the same for humics-mediated 2,4-D transformation. This study demonstrated the important role of humics reduction in the redox cycle of iron and the environmental transformation of chlorinated organic pollutants under alkaline conditions.

## Experimental procedures

### Materials

Humics analogues (AQDS, AQS and AQC), FA and HA, of chemical grade were purchased from Sigma-Aldrich (Tokyo, Japan). 2,4-D, of analytical grade, was also purchased from Sigma-Aldrich. All of the chemicals were used as received, without further purification. α-FeOOH was synthesized according to the procedures of Li and colleagues (2008). Mineral salts medium (MSM) used for anaerobic incubation was prepared as previously described (Wu *et al*., 2011).

*Corynebacterium humireducens* MFC-5 was isolated from the anode of a wastewater-fed MFC that was continuously operated at a pH of 10.0. The strain MFC-5 is active for anaerobic reduction of AQDS, with lactate, formate, acetate, ethanol, or sucrose as electron donor (Wu *et al*., 2011). Resting cell suspension of MFC-5 was used in this study. The suspension was aerobically prepared in Luria–Bertani (LB) medium at 30°C, and then harvested at the late log phase by centrifugation (8000 g at 4°C for 10 min). Pellets was then washed twice and re-suspended in sterilized MSM (pH 9.0).

### Batch reduction experiments

Strict anaerobic techniques and sterile conditions were used throughout all the incubation experiments. MSM was adjusted to pH 9.0 using 0.1 mmol l^−1^ NaOH, and maintained with 20 mmol l^−1^ carbonate buffer (pH 9.0). The serum bottles (25.2 ml) contained alkaline MSM that was sterilized by autoclaving at 115°C for 20 min. Other components (i.e. sucrose, quinones/humics, Fe(III), 2,4-D or cell suspension) were added from their corresponding stock solutions after MSM was cooled to ambient temperature. Then the bottles were purged with O_2_-free N_2_/CO_2_ (80/20 v/v) for 15 min, sealed with butyl-rubber stoppers and crimped with aluminum caps. All bottles were incubated in the dark at 30°C, which was optimal for the microbial growth of strain MFC-5. All the treatments were conducted in triplicate.

In the experiments testing alternative electron acceptors for MFC-5 anaerobic metabolisms, each bottle contained 1 × 10^7^ cells ml^−1^, 20 ml MSM (pH 9.0) with 5 mmol l^−1^ of sucrose (electron donor) and one of the following substrates as electron acceptor: 1 mmol l^−1^ of AQDS, AQS, AQC, FA or 200 mg l^−1^ of HA; 5 mmol l^−1^ α-FeOOH and 180 μmol l^−1^ of 2,4-D. Two control assays were performed under the same conditions: an abiotic set without bacterial cells and a biotic set without the addition of sucrose.

For studying the influence of humics reduction by the strain MFC-5 on the anaerobic reduction of iron(III) oxides and 2,4-D under alkaline conditions, four batch experiments were conducted: (i) MSM + (α-FeOOH or 2,4-D) + sucrose + (AQDS, AQS, AQC, FA or HA), (ii) MSM + (α-FeOOH or 2,4-D) + sucrose + (AQDS, AQS, AQC, FA or HA) + dead cells of MFC-5, (iii) MSM + (α-FeOOH or 2,4-D) + sucrose + MFC-5, and (iv) MSM + (α-FeOOH or 2,4-D) + sucrose + (AQDS, AQS, AQC, FA or HA) + MFC-5. Set (i) and (ii) were abiotic controls to evaluate the abiotic transformation of α-FeOOH and 2,4-D by sucrose, humics and dead cells, and set (iii) served as a biotic control. The concentrations of each component were: 5 mmol l^−1^ Fe(III), 180 μmol l^−1^ 2,4-D, 0.5 mmol l^−1^ AQDS/AQS/AQC/FA, 200 mg l^−1^ HA, and 1 × 10^7^ cells ml^−1^.

### Analysis methods

Triplicate bottles were sacrificed for chemical analysis. The concentration of AH_2_QDS, AH_2_QS and AH_2_QC was quantified by UV-Vis spectrophotometer (TU1800-PC, Beijing). As shown in Fig. S3, the specific wavelengths of AH_2_QDS, AH_2_QS and AH_2_QC at pH 9.0 were 408, 397 and 380 nm respectively. FAred and HAred were determined with Fe(III)-citrate as previously described (Lovley *et al*., 2000).

Total Fe(II), including dissolved and sorbed Fe(II), was quantified photometrically at 510 nm after being extracted using 0.5 mol l^−1^ HCl for 1.5 h and reacting with 1,10-phenanthroline. Dissolved Fe(II) was determined by removing the mineral and sorbed Fe(II) from the aqueous phase using a 0.22 mm syringe filter and then assaying the filtrate using the colorimetric method (Li *et al*., 2010). The difference between the total and dissolved Fe(II) was defined as sorbed Fe(II). The concentration of 2,4-D and its intermediates was analysed by HPLC (Waters 1527/2487) as described by Wu and colleagues (2010).

FT-IR (Vector 33, Bruker, Germany) was used to determine the functional groups of humics. The interpretations of the FT-IR spectra were based on the literature of D'Orazio and Senesi (2009). CV was used to evaluate the redox behaviour of 2,4-D (180 μmol l^−1^), quinones (0.5 mmol l^−1^) and 2,4-D/quinones. Voltammograms were obtained using a potentiostat (CHI605C, Shanghai Chenhua, China) and a three-electrode cell (20 mmol l^−1^ carbonate buffer of pH 9.0; 500 mmol l^−1^ NaClO_4_ electrolyte, 3 mm diameter Pt disk working electrode, Hg wire counter electrode, Ag/AgCl reference electrode) under continuous N_2_ bubbling. Tested solutions were deoxygenated by purging with O_2_-free N_2_ gas for 30 min before CVs measurement, and the scan data were recorded from −0.8 to 0 V at a scan rate of 50 mV s^−1^ at intervals. Chronoamperometry was employed to determine the EAC of humics by applying fixed positive or negative potentials to a working electrode in a conventional three-electrode cell (Yuan *et al*., 2011).

## Conflict of interest

None declared.

## Supporting information

**Fig. S1.** FT-IR spectra of five types of humics or quinones (HA, FA, AQDS, AQS, AQC). **Fig. S2.** Proposed mechanism of humics-mediated reduction of α-FeOOH and 2,4-D in the system of α-FeOOH/2,4-D+MFC-5+Humics+sucrose under alkaline conditions. **Fig. S3.** UV-Vis absorption spectra for 0.5 mmol l^–1^ quinones (AQDS, AQS and AQC) or 0.5 mM hydroquinones (AH_2_QDS, AH_2_QS, and AH_2_QC) at pH 9.0. Specific wavelengths of AH_2_QDS, AH_2_QS, and AH_2_QC at pH 9.0 were 408 nm, 397 nm and 380 nm respectively.
