# Supplementary material for: Humic substance-mediated reduction of iron(III) oxides and degradation of 2,4-D by an alkaliphilic bacterium, Corynebacterium humireducens MFC-5
Source: Microb Biotechnol. 2012 Dec 6;6(2):141–9. doi: 10.1111/1751-7915.12003 (PMC3917456; doi:10.1111/1751-7915.12003)
Supplement: Supplementary file 1 — Fig. S1. FT-IR spectra of five types of humics or quinones (HA, FA, AQDS, AQS, AQC). Fig. S2. Proposed mechanism of humics-mediated reduction of α-FeOOH and 2,4-D in the system of α-FeOOH/2,4-D+MFC-5+Humics+sucrose under alkaline conditions. Fig. S3. UV-Vis absorption spectra for 0.5 mmol l–1 quinones (AQDS, AQS and AQC) or 0.5 mM hydroquinones (AH2QDS, AH2QS, and AH2QC) at pH 9.0. Specific wavelengths of AH2QDS, AH2QS, and AH2QC at pH 9.0 were 408 nm, 397 nm and 380 nm respectively. [file mbt20006-0141-sd1.doc]

**Figure S1**

**Figure S2**

**α-FeOOH/2,4-D**

**Sucrose**

**MFC-5**

**Quinone/Humics**

**+e**

**-e**

**Hydroquinone/**

**Reduced humics**

**(i) Biotic process**

**-e**

**+e**

**Abiotic process**

**Mineral-bound Fe(II) species/**

**2,4-D transformation product**

**Organic acids**

**/CO_2_**

**Figure S3**
